# Rural-urban differences in food insecurity and associated cognitive impairment among older adults: findings from a nationally representative survey

**DOI:** 10.1186/s12877-022-02984-x

**Published:** 2022-04-06

**Authors:** Shobhit Srivastava, T. Muhammad

**Affiliations:** International Institute for Population Sciences, Mumbai, Maharashtra 400088 India

**Keywords:** Food insecurity, Cognitive impairment, Older adults, India

## Abstract

**Introduction:**

Due to rapid urbanization, Covid-19 pandemic and increasing food prices, a higher rate of food insecurity has been observed in recent years in India. Thus, we aim to study the prevalence of food insecurity among older Indian adults and the association of food insecurity as a modifiable risk factor with late-life cognitive impairment.

**Method:**

Data for this study were obtained from the recent release of the Longitudinal Ageing Study in India (2017–18). The total sample size for the study was 31,464 older adults aged 60 years and above. Cognitive functioning was measured through five broad domains (memory, orientation, arithmetic function, executive function, and object naming) adapted from the cognitive module of the US Health and Retirement Study (HRS). Descriptive statistics along with cross-tabulation were presented in the study. Additionally, multivariable logistic regression analysis was used to fulfil the objectives of the study.

**Results:**

It was found that 7.7% of older adults in rural areas reduced their size of meals due to unavailability (urban, 3.2%), 41.2% of them did not eat enough food of their choice (urban, 38.3%), 6.9% were hungry but did not eat food (urban, 2.6%), 5.0% did not eat for whole day (urban, 2.2%), and 6.9% lost weight due to lack of food in their household (urban, 2.9%). It was found that older adults who did not have enough food of their choice had significantly higher odds [AOR: 1.24; CI: 1.14, 1.35] of suffering from cognitive impairment in reference to their counterparts. Similarly, the older adults who were hungry but did not eat were 30% [AOR: 1.30; CI: 1.02, 1.73] more likely to suffer from cognitive impairment in reference to their counterparts. Interaction model revealed that older adults who had food insecurity in rural areas had higher odds of cognitive impairment than older adults who had food insecurity in urban areas.

**Conclusion:**

The findings of the study highlight that the food security status in older adults may bring about greater challenges due to their limited economic resources. Interventions focusing on food security may have unintended positive impacts on late-life mental wellbeing as the older age is associated with higher cognitive deficits.

**Supplementary Information:**

The online version contains supplementary material available at 10.1186/s12877-022-02984-x.

## Introduction

The proportion of the older population in India has risen from 5.6% in 1961 to 8.5% in 2011 [1], and the projection is made that the country will host over 319 million older adults aged 60 years and above which is nearly 20% of the total population [2]. Apart from such demographic changes, the country also faces higher poverty and inadequate food which result in worse physical and mental health outcomes especially in vulnerable populations including older individuals [3, 4].

Food insecurity refers to the unavailability of nutritionally adequate and safe foods or inability to acquire foods in socially acceptable ways [5]. Recent projections show that despite some progress, the world is not on track to achieve Sustainable Development Goal 2, Zero Hunger by 2030 [6, 7]. More than one in 9 of the world population do not get enough to eat and the prevalence of undernourishment is considerably high in developing countries [8]. Evidence also suggests that the food security status of the most vulnerable population groups is likely to deteriorate further [9, 10]. On the other hand, the linkage between food security and health conditions has been well-documented among different populations [11, 12], including older adults [13]. Current research suggests that household food insecurity is associated with adverse mental health outcomes across the lifespan [14, 15]. Also, there is increasing evidence that food insecurity is associated with decline in cognitive abilities among aging populations [16–19].

A growing body of literature suggests that food insecurity may magnify the existing socioeconomic disparities within populations which in turn may increase the risk of those who are vulnerable and influence their mental wellbeing [20]. The inability to intake food due to functional limitations and health problems may also result in higher risk of food insecurity among older population consequently leading to a decreased mental health status [21, 22]. However, the exact mechanisms linking food insecurity and cognitive impairment are not yet clear. A couple of studies suggest that the stress resulting from food insecurity may increase the risk of declining cognitive abilities. For example, elevated cortisol can lead to changes in the functions of brain and subsequent decline in cognitive abilities [23]. It has also been hypothesized that the increasing age being a risk factor, food insecurity may increase the risk of cognitive decline via stress, depression, or poor nutritional intake [24]. Poor diet on the other hand, has been associated with increased risk of cognitive decline and several vitamins have been shown to have a protective effect against dementia [25].

However, despite the fact that food insecurity is more common in poor resource settings, there are only few studies specifically on the association between food insecurity and cognitive impairment in low- and middle-income countries [17]. Also, due to rapid urbanization, Covid-19 pandemic, and increasing food prices, a higher rate of food insecurity has been observed in recent years in India [26–28]. Thus, we aim to study the prevalence of food insecurity among older Indian adults and the association of food insecurity as a modifiable risk factor with late-life cognitive impairment. The study hypothesized that there are rural-urban differentials in food insecurity and associated cognitive dysfunction among older adults in India.

## Methods

### Data

Data for this study were obtained from the recent release of Longitudinal Ageing Study in India (LASI) wave 1 [29]. LASI is a full-scale national survey of scientific investigation of the health, economic, and social determinants and consequences of population aging in India, conducted in 2017–18. The LASI is a nationally representative survey of over 72,000 adults aged 45 and above across all states and union territories (UTs) of India [29]. The main objective of the survey is to study the health status and the social and economic well-being of older adults in India. The LASI adopted a multistage stratified area probability cluster sampling design to arrive at the eventual units of observation: older adults age 45 and above and their spouses irrespective of age. The survey adopted a three-stage sampling design in rural areas and a four-stage sampling design in urban areas. In each state/UT, the first stage involved the selection of Primary Sampling Units (PSUs), that is, sub-districts (Tehsils/Talukas), and the second stage involved the selection of villages in rural areas and wards in urban areas in the selected PSUs [29]. In rural areas, households were selected from selected villages in the third stage. However, sampling in urban areas involved an additional stage. Specifically, in the third stage, one Census Enumeration Block (CEB) was randomly selected in each urban area [29]. In the fourth stage, households were selected from this CEB. The detailed methodology, with the complete information on the survey design and data collection, was published in the survey report and elsewhere [29, 30]. The present study is conducted on eligible respondents aged 60 years and above. The total sample size for the study was 31,464 older adults aged 60 years and above. The sample from rural areas was 20,725 and from urban areas was 10,739 older adults.

### Variable description

#### Outcome variable

Cognitive functioning in the LASI survey was measured through five broad domains (memory, orientation, arithmetic function, executive function, and object naming) adapted from the cognitive module of the U S Health and Retirement Study (HRS). The description of these tests is given in the supplementary material(Table 1). The cognitive impairment in the current study is based on those domains with different cognitive measures including: immediate (0–10 points) and delayed word recall (0–10 points); orientation related to time (0–4 points), and place (0–4 points); arithmetic ability based on serial 7 s (0–5 points), computation (0–2) and backward counting from 20 (0–2 points); executive functioning based on paper folding (0–3) and pentagon drawing (0–1); and object naming (0–2). The overall score ranges between 0 and 43, and a higher score indicates better cognitive functioning. In our study, the respondents who received assistance during the cognition module were excluded from the analysis. The lowest 10th percentile is used as a proxy measure of poor cognitive functioning or cognitive impairment [29].

**Table 1:** Socio-economic profile of older adults in India, 2017–18

Background characteristics	Rural		Urban	
	Sample	Percentage	Sample	Percentage
**Individual characteristics**				
**Age**				
Young-old	12,139	58.6	6268	58.4
Old-old	6169	29.8	3354	31.2
Oldest-old	2417	11.7	1117	10.4
**Sex**				
Male	10,045	48.5	4835	45.0
Female	10,680	51.5	5904	55.0
**Education**				
Not educated/primary not completed	15,986	77.1	4937	46.0
Primary	2069	10.0	1511	14.1
Secondary	1988	9.6	2598	24.2
Higher	682	3.3	1693	15.8
**Working status**				
Working	7341	35.4	2106	19.6
Retired/currently not working	8774	42.3	4719	43.9
Never worked	4610	22.2	3913	36.4
**Marital status**				
Currently married	13,017	62.8	6315	58.8
Widowed	7280	35.1	4162	38.8
Others	427	2.1	262	2.4
**Living arrangement**				
Living alone	1311	6.3	444	4.1
Living with spouse	4455	21.5	1883	17.5
Living with children and spouse	13,708	66.1	7873	73.3
Living with others.	1251	6.0	539	5.0
**Social participation**				
No	19,844	95.8	10,197	95.0
Yes	881	4.3	542	5.1
**Physical activity**				
Frequent	3980	19.2	1610	15.0
Rarely	3101	15.0	813	7.6
Never	13,644	65.8	8317	77.4
**Health-related factors**				
**Depression**				
No	18,271	90.4	9774	93.7
Yes	1945	9.6	662	6.3
**Self-rated health**				
Good	9969	49.1	6000	57.1
Poor	10,326	50.9	4516	42.9
**Difficulty in ADL**				
No	15,625	75.4	8190	76.3
Yes	5100	24.6	2549	23.7
**Difficulty in IADL**				
No	10,015	48.3	6262	58.3
Yes	10,710	51.7	4477	41.7
**Morbidity**				
No morbidity	10,926	52.7	3558	33.1
1	5840	28.2	3380	31.5
2+	3959	19.1	3800	35.4
**Household factors**				
**MPCE quintile**				
Poorest	4446	21.5	2396	22.3
Poorer	4608	22.2	2197	20.5
Middle	4375	21.1	2207	20.6
Richer	3932	19.0	2117	19.7
Richest	3364	16.2	1822	17.0
**Religion**				
Hindu	17,309	83.5	8497	79.1
Muslim	2021	9.8	1604	14.9
Christian	623	3.0	269	2.5
Others	772	3.7	369	3.4
**Caste**				
Scheduled Caste	4572	22.1	1220	11.4
Scheduled Tribe	2125	10.3	325	3.0
Other Backward Class	9213	44.5	5056	47.1
Others	4815	23.2	4139	38.5
**Region**				
North	2655	12.8	1293	12.0
Central	4920	23.7	1533	14.3
East	5678	27.4	1573	14.7
Northeast	691	3.3	226	2.1
West	2898	14.0	2662	24.8
South	3883	18.7	3451	32.1
**Total**	20,725	100.0	10,739	100.0

#### Explanatory variable

The main explanatory variables were derived from food security section of the LASI dataset. The five questions which were related to food security among older adults in the LASI survey were adapted from similar items established in food security questionnaires of the U.S. Household Food Security Survey Module (HFSSM) adult scale [31], and the items are validated in Indian settings [32]. The items are:

1. In the last 12 months, did you ever reduce the size of your meals or skip meals because there was not enough food at your household? The variable generated using this question was “reduced the size of meals” and it was coded as 0 “no” and 1 “yes”.
2. In the last 12 months, did you eat enough food of your choice? Please exclude fasting/food related restrictions due to religious or health related reason. The variable generated using this question was “did not eat food of once choice” and it was coded as 0 “no” and 1 “yes”.
3. In the last 12 months, were you hungry but didn’t eat because there was not enough food at your household? Please exclude fasting/food related restrictions due to religious or health related reasons. The variable generated using this question was “hungry but did not eat” and it was coded as 0 “no” and 1 “yes”.
4. In the past 12 months did you ever not eat for a whole day because there was not enough food at your household? Please exclude fasting/food related restrictions due to religious or health related reasons. The variable generated using this question was “did not eat for a whole day” and it was coded as 0 “no” and 1 “yes”.
5. Do you think that you have lost weight in the last 12 months because there was not enough food at your household? The variable generated using this question was “lost weight due to lack of food” as it was coded as 0 “no” and 1 “yes”.

Further, due to their nature of binary response (no/yes), these questions were intended to be variables indicative of various dimensions of food insecurity and did not constitute a comprehensive food security measure. Thus, food insecurity in the current study was defined as affirmative response to any of these five questions and was analysed separately for each questions.

The main stratifying variable for the present study was place of residence which was coded as rural and urban.

#### Individual factors

A couple of socio-demographic and behavioural factors were included in the analysis according to the abovementioned literature. Age was coded as young old (60–69 years), old-old (70–79 years), and oldest-old (80+ years). Sex was coded as male and female. Educational status was coded as no education/primary not completed, primary, secondary and higher. Working status was coded as currently working, retired/currently not working, and never worked [33]. Marital status was coded as currently married, widowed and others. Others included divorced/separated/never married. Living arrangement was coded as living alone, living with spouse, living with spouse and children and living with others. Social participation was coded as no and yes. Social participation was measured through the question “Are you a member of any of the organizations, religious groups, clubs, or societies? The response was coded as no and yes. Physical activity status was coded as frequent (every day), rare (more than once a week, once a week, one to three times in a month), and never. The question through which physical activity was assessed was “How often do you take part in sports or vigorous activities, such as running or jogging, swimming, going to a health centre or gym, cycling, or digging with a spade or shovel, heavy lifting, chopping, farm work, fast bicycling, cycling with loads”? [29].

#### Health-related factors

Several health-related variables were selected as possible confounders in the analysis as per the literature, including depression [34], self-rated health, functional limitations [35] and morbidity [36]. The probable major depression among the older adults with symptoms of dysphoria, calculated using the CIDI-SF (Short Form Composite International Diagnostic Interview) those with a score of 3 or more were considered as depressed. This scale estimates a probable psychiatric diagnosis of major depression and has been validated in field settings and widely used in population-based health surveys [29, 37]. Self-rated health was coded as good which includes excellent, very good, and good whereas poor includes fair and poor [35]. Difficulty in activities of daily living (ADL) was coded as no and yes. ADL is a term used to refer to normal daily self-care activities (such as movement in bed, changing position from sitting to standing, feeding, bathing, dressing, grooming, personal hygiene, etc.) The ability or inability to perform ADLs is used to measure a person’s functional status, especially in the case of people with disabilities and the ones in their older ages [33]. Difficulty in Instrumental ADL (IADL) was coded as no and yes. These tasks are necessary for independent functioning in the community. Respondents were asked if they were having any difficulties that were expected to last more than3 months, such as preparing a hot meal, shopping for groceries, making a telephone call, taking medications, doing work around the house or garden, managing money (such as paying bills and keeping track of expenses), and getting around or finding an address in unfamiliar places [38]. Morbidity was coded as no morbidity, 1 and 2+ [38].

#### Household factors

The following household or community-related variables were also added in the analysis as control variables. The monthly per capita consumption expenditure (MPCE) was assessed using household consumption data. Sets of 11 and 29 questions on the expenditures on food and non-food items, respectively, were used to canvas the sample households. Food expenditure was collected based on a reference period of 7 days, and non-food expenditure was collected based on reference periods of 30 days and 365 days. Food and non-food expenditures have been standardized to the 30-day reference period. The MPCE is computed and used as the summary measure of consumption [29]. The variable was then divided into five quintiles i.e., from poorest to richest. Religion was coded as Hindu, Muslim, Christian, and Others. Caste was recoded as Scheduled Tribe (ST), Scheduled Caste (SC), Other Backward Class (OBC), and others. The SC includes a group of the population that is socially segregated and financially/economically by their low status as per Hindu caste hierarchy. The STs and SCs are among the most disadvantaged and discriminated socio-economic groups in India. The OBC is the group of people who were identified as “educationally, economically and socially backward”. The “other” caste category is identified as having higher social status [39]. The regions of India were coded as North, Central, East, Northeast, West, and South.

### Statistical analysis

Descriptive statistics along with cross-tabulation were presented in the study. Proportion test was used to evaluate the significance level of differences in cognitive impairment among older adults from rural and urban place of residence [40]. Additionally, multivariable binary logistic regression analysis [41] was used to find the association between the outcome variable (cognitive impairment) and food security measures among older adults in India.

The binary logistic regression model is usually put into a more compact form as follows:

$$\mathrm{Logit}\ \left[\mathrm{P}\left(\mathrm{Y}=1\right)\right]={\beta}_0+\beta \ast X$$

The parameter *β*_0_ estimates the log odds of cognitive impairment for the reference group, while *β* estimates the maximum likelihood, the differential log odds of cognitive impairment associated with a set of predictors X, as compared to the reference group. Variance inflation factor (VIF) was generated in STATA 14 [42] to check the multicollinearity and it was found that there was no evidence of multicollinearity in the variables used [43, 44].

Moreover, interaction effects [36] were observed for food security variables and place of residence with cognitive impairment among older adults. Model-3 to model-7 in Fig. 3b provide adjusted estimates for interaction effects.

## Results

Table 1 represents the socio-economic profile of older adults. It was found that about 77% of older adults were not educated in rural areas whereas in urban areas the percentage was 46.0%. There is higher percentage of older adults never worked in urban areas (36.4%) than in rural areas (22.2%). Higher percentage of older adults in rural areas were living alone (6.3%) than in urban areas (4.1%). A proportion of 65.8% of older adults in rural areas did not involve in physical activity whereas the percentage was 77.4% in urban areas. Higher percentage of older adults in rural areas (9.6%) suffered from depression than in urban areas (6.3%). Higher percentage of older adult in rural areas (50.9%) reported poor SRH in reference to older adults in urban areas (42.9%). About 51.7% of older adults in rural areas had difficulty in IADL in reference to 41.7% in urban areas. Higher percentage of older adult from urban areas suffered from two or more morbidities (35.4%) in comparison to older adults in rural areas (19.1%).

**Fig. 1:**
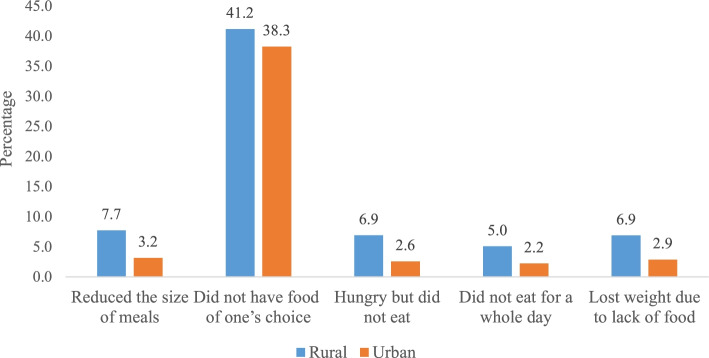
Percentage distribution of older adults by food insecurity measures.

Figure 1 reveals that a higher percentage of older adults in rural areas (7.7%) reduced their size of meals because there was not enough food in their household (urban, 3.2%). Higher percentage of older adults in rural areas (41.2%) did not eat enough food of their choice because there was not enough food in their household (urban, 38.3%). Similarly, higher percentage of older adults in rural areas (6.9%) were hungry but did not eat food because there was not enough food in their household (urban, 2.6%). Higher percentage of older adults in rural areas (5.0%) did not eat for whole day because there was not enough food in their household (urban, 2.2%). Higher percentage of older adults in rural areas (6.9%) lost weight due to lack of food in their household (urban, 2.9%).

**Fig. 2:**
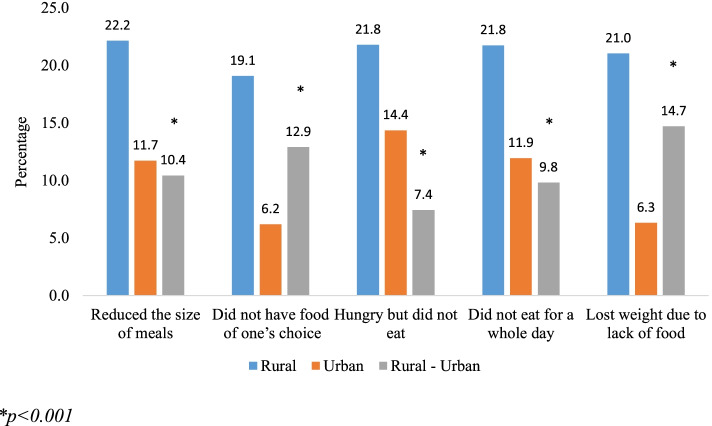
Percentage of older adults suffering from cognitive impairment by food insecurity measures and place of residence.

Figure 2 represents the percentage of older adults suffering from cognitive impairment by place of residence in India. It was found that in all categories older adults from rural residence had significantly higher percentage of cognitive impairment.

**Fig. 3:**
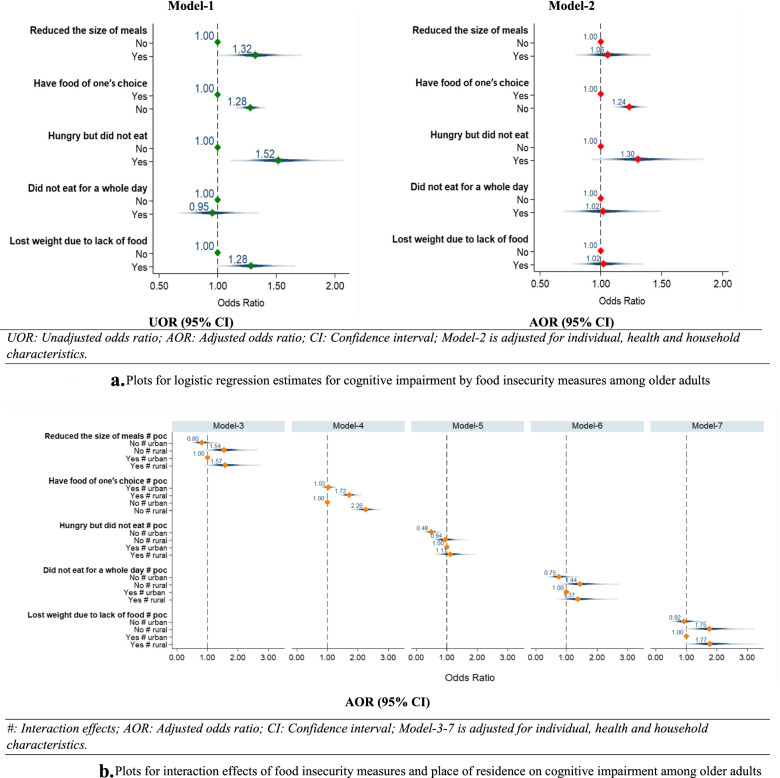
**a** Plots for logistic regression estimates for cognitive impairment by food insecurity measures among older adults AOR (95% CI). #: Interaction effects; AOR: Adjusted odds ratio; CI: Confidence interval; Model-3-7 is adjusted for individual, health and household characteristics. **b** Plots for interaction effects of food insecurity measures and place of residence on cognitive impairment among older adults

Figure 3a represents plots for logistic regression estimates for cognitive impairment among older adults in India. Model-1 which reveals unadjusted estimates and it was found that older adults who reduced the size of the meal had 32% [UOR: 1.32; CI: 1.09,1.63] significantly higher likelihood to suffer from cognitive impairment than older adults who did not reduce their meal size. Older adults who did not have enough food of their choice had higher odds [UOR:1.28; CI: 1.19, 1.39] for cognitive impairment than older adults who had enough food of their choice. Older adults who reported that they did not eat when they were hungry had 52% [UOR: 1.52; CI: 1.21, 1.95]; significantly higher likelihood to suffer from cognitive impairment than their counterpart. Older adults who reported that they lost weight due to lack of food had higher odds [UOR: 1.28; CI: 1.04, 1.54] for cognitive impairment in reference to their counterparts. Model-2 provide the adjusted estimates and it was found that older adults who did not have enough food of their choice had significantly higher odds [AOR: 1.24; CI: 1.14, 1.35] to suffer from cognitive impairment in reference to their counterparts. Similarly, older adults who were hungry but did not eat food were 30% [AOR: 1.30; CI: 1.02, 1.73] more likely to suffer from cognitive impairment in reference to their counterparts. The adjusted estimates along with other socioeconomic and health-related covariates can be found in Table-S2 (supplementary file).

Figure 3b presents the plots for interaction effects food insecurity measures and place of residence on cognitive impairment among older adults. Estimates for model-3 revealed that older adults who reduced their size of meal in rural areas were 61% [AOR: 1.57; CI: 1.06, 2.46] significantly more likely to suffer from cognitive impairment in reference to the older adults who reduced their size of meal in urban areas. Similarly, older adults who did not eat enough food of their choice in rural areas had significantly higher odds [AOR: 2.26; CI: 1.91, 2.66] for cognitive impairment in comparison to older adults who did not eat enough food of their choice in urban areas. Older adults who were hungry or did not eat for a whole day in rural areas had higher odds for cognitive impairment in reference to older adults who were hungry or did not eat for a whole day in urban areas respectively. However, the estimates were not significant. Older adults who lost their weight due to lack of food in rural areas were 77% [AOR: 1.77: 1.08, 2.88] significantly more likely to suffer from cognitive impairment in reference to older adults who lost weight due to lack of food in urban areas.

## Discussion

Due to increasing importance of food environment, there is a growing attention in the recent research towards the problems and challenges of hunger and the measures of food availability. Accordingly, a variety of methodologies including objective and self-perceived measures have been used to determine the availability and accessibility of food [45–47]. The current study using the respondent-based measures of food insecurity found a substantially higher proportion of older population experiencing a reduced or lack of food availability (particularly food of one's choice) and subsequent weight loss.

In the current sample of older population, most of the indicators of food insecurity were positively associated with cognitive impairment, independent of socio-demographic factors. The findings corroborate a previous study in which the authors found that food insecurity was associated with lower levels cognitive functioning among older adults, and that at more extreme levels of food insecurity, the magnitude of the association was greater [48]. Moreover, studies suggest that with lower intake of food, multiple processes in human bodies such as protein folding, degradation, and nutrient-sensing may get damaged [24, 49]. In the present analyses, the significant associations of multiple measures of food insecurity with cognitive impairment were observed even after adjusting for individual, health and household factors, suggesting food insecurity as an important social determinant that may contribute to cognitive decline in older ages. The results are also consistent with studies suggesting that food insecurity and hunger being biological and psychosocial stressors may potentially increase psycho-emotional problems particularly during older age [15].

Compared to older adults living in urban areas, those living in rural areas are found to be experiencing more health problems in multiple studies due to their inadequate access to health care services and resources [50–52]. Rural living older individuals may also face physical immobility, lack of cooking skills, and lack of or limited supports for food and non-food products that can exacerbate the association between food insecurity and several mental disorders [53, 54]. In agreement with these findings, our analysis has shown that rural living older persons were more likely to be cognitively impaired if they were food insecure. Notably, among various measures of food insecurity in the current study, an important finding is that limited diet quality indicated by "did not have food of one's choice" (than skipping/reducing meals, being hungry, not eating, and weight loss due to lack of food) was significantly associated with cognitive impairment, especially in those residing in rural areas. This may be attributed to the earlier studies that have reported that older population living in rural areas have limited access to food stores and wish away from their healthy food choices [55–57]. Concordantly, several randomized clinical trials have shown that supplementation of nutritious foods may improve cognitive function in older individuals [58–60] and in rural areas in particular [61]. The finding may also be attributed to the limited food intake due to older individuals' functional limitations and other health problems. However, high intake of carbohydrate and macronutrients has been associated with higher risk of cognitive impairment [62]. Hence, further research is needed to explore the role of older individuals' food choices and availability of particular quality food items that enhance the cognitive abilities in relation to food insecurity among residents in rural settings.

According to different rounds of National Sample Surveys, although food is accounted for the highest household expenditure, food expenditure on older adults has been decreasing over the time [63]. The urban poor are more likely to spend 60% more of their earnings on food than the rural poor [26]. However, in the present study, the results of interactive effects show that people aged ≥60 years not eating food of their choice and residing in urban areas had 2.26 times higher odds for cognitive impairment compared with those who had their choice of food and resided in urban areas. This needs to be further investigated with follow-up rounds of LASI survey. Considering the current findings, long-term programs and multifaceted initiatives are needed to prevent food insecurity and address the poverty-related poor cognitive function among older population. The policies should also focus on connecting food insecure households especially those in rural areas to existing social services such as Public Distribution System in India as part of efforts in mitigating the late-life cognitive dysfunction and ensuring healthy aging.

The study had certain limitations. The data were cross-sectional in nature therefore how change in food insecurity lead to change in cognitive impairment in later lives could not be estimated. However, the study has its own strengths. Firstly, the cognitive impairment variable was measured using the standard scales. Secondly, the data is latest, therefore provides the current scenario among older adults in India. Finally, the data is first of its kind collected on pan India level with comprehensive information on aging population in the country.

## Conclusion

The findings of the study highlight that the food security status in older adults may bring about greater challenges due to their limited economic resources. Interventions focusing on food security may have unintended positive impacts on late-life mental wellbeing as the older age is associated with higher cognitive deficits.

## Supplementary Information

**Additional file 1.**

## Data Availability

The study uses a secondary data which is available on reasonable request through https://www.iipsindia.ac.in/content/lasi-wave-i.
