# Supplementary material for: Rural-urban differences in food insecurity and associated cognitive impairment among older adults: findings from a nationally representative survey
Source: BMC Geriatr. 2022 Apr 6;22:287. doi: 10.1186/s12877-022-02984-x (PMC8985064; doi:10.1186/s12877-022-02984-x)
Supplement: Supplementary file 1 — Additional file 1. [file 12877_2022_2984_MOESM1_ESM.docx]

| **Table-S1.** Logistic regression analysis for cognitive impairment among older adults in India, 2017-18 | | | | | | | |
| --- | --- | --- | --- | --- | --- | --- | --- |
| **Background characteristics** | **Model-1** | **Model-2** | **Model-3** | **Model-4** | **Model-5** | **Model-6** | **Model-7** |
|  | **UOR (95% CI)** | **AOR (95% CI)** | **AOR (95% CI)** | **AOR (95% CI)** | **AOR (95% CI)** | **AOR (95% CI)** | **AOR (95% CI)** |
| **Food security factors** |  |  |  |  |  |  |  |
| **Reduced the size of meals** |  |  |  |  |  |  |  |
| No | Ref. | Ref. |  |  |  |  |  |
| Yes | 1.32*(1.09,1.63) | 1.06(0.86,1.34) |  |  |  |  |  |
| **Did not have food of once choice** |  |  |  |  |  |  |  |
| No | Ref. | Ref. |  |  |  |  |  |
| Yes | 1.28*(1.19,1.39) | 1.24*(1.14,1.35) |  |  |  |  |  |
| **Hungry but did not eat** |  |  |  |  |  |  |  |
| No | Ref. | Ref. |  |  |  |  |  |
| Yes | 1.52*(1.21,1.95) | 1.30*(1.02,1.73) |  |  |  |  |  |
| **Did not eat for a whole day** |  |  |  |  |  |  |  |
| No | Ref. | Ref. |  |  |  |  |  |
| Yes | 0.95(0.74,1.25) | 1.02(0.77,1.38) |  |  |  |  |  |
| **Lost weight due to lack of food** |  |  |  |  |  |  |  |
| No | Ref. | Ref. |  |  |  |  |  |
| Yes | 1.28*(1.04,1.54) | 1.02(0.80,1.23) |  |  |  |  |  |
| **Individual factors** |  |  |  |  |  |  |  |
| **Age** |  |  |  |  |  |  |  |
| Young-old |  | Ref. |  |  |  |  |  |
| Old-old |  | 1.60*(1.46,1.77) |  |  |  |  |  |
| Oldest-old |  | 2.94*(2.58,3.36) |  |  |  |  |  |
| **Sex** |  |  |  |  |  |  |  |
| Male |  | Ref. |  |  |  |  |  |
| Female |  | 2.13*(1.91,2.38) |  |  |  |  |  |
| **Education** |  |  |  |  |  |  |  |
| Not educated/primary not completed |  | 24.81*(11.73,52.48) |  |  |  |  |  |
| Primary |  | 4.15*(1.9,9.05) |  |  |  |  |  |
| Secondary |  | 2.05(0.91,4.61) |  |  |  |  |  |
| Higher |  | Ref. |  |  |  |  |  |
| **Working status** |  |  |  |  |  |  |  |
| Working |  |  |  |  |  |  |  |
| Retired |  | 1.18*(1.04,1.33) |  |  |  |  |  |
| Not working |  | 1.29*(1.13,1.48) |  |  |  |  |  |
| **Marital status** |  |  |  |  |  |  |  |
| Currently married |  | Ref. |  |  |  |  |  |
| Widowed |  | 1.39*(1.24,1.55) |  |  |  |  |  |
| Others |  | 1.35*(1.01,1.8) |  |  |  |  |  |
| **Living arrangement** |  |  |  |  |  |  |  |
| Living alone |  | Ref. |  |  |  |  |  |
| Living with spouse |  | 1.16(0.94,1.44) |  |  |  |  |  |
| Living with children and spouse |  | 0.99(0.83,1.18) |  |  |  |  |  |
| Living with others. |  | 1.26*(1,1.57) |  |  |  |  |  |
| **Social participation** |  |  |  |  |  |  |  |
| No |  | 1.57*(1.24,1.99) |  |  |  |  |  |
| Yes |  | Ref. |  |  |  |  |  |
| **Physical activity** |  |  |  |  |  |  |  |
| Frequent |  | Ref. |  |  |  |  |  |
| Rarely |  | 1.06(0.89,1.26) |  |  |  |  |  |
| Never |  | 1.31*(1.14,1.5) |  |  |  |  |  |
| **Health factors** |  |  |  |  |  |  |  |
| **Depression** |  |  |  |  |  |  |  |
| No |  | Ref. |  |  |  |  |  |
| Yes |  | 1.08(0.93,1.26) |  |  |  |  |  |
| **Self-rated health** |  |  |  |  |  |  |  |
| Good |  | Ref. |  |  |  |  |  |
| Poor |  | 1.33*(1.22,1.46) |  |  |  |  |  |
| **Difficulty in ADL** |  |  |  |  |  |  |  |
| No |  | Ref. |  |  |  |  |  |
| Yes |  | 1.38*(1.24,1.53) |  |  |  |  |  |
| **Difficulty in IADL** |  |  |  |  |  |  |  |
| No |  | Ref. |  |  |  |  |  |
| Yes |  | 1.37*(1.24,1.51) |  |  |  |  |  |
| **Morbidity** |  |  |  |  |  |  |  |
| No morbidity |  | Ref. |  |  |  |  |  |
| 1 |  | 0.81*(0.73,0.9) |  |  |  |  |  |
| 2+ |  | 0.73*(0.65,0.82) |  |  |  |  |  |
| **Household factors** |  |  |  |  |  |  |  |
| **MPCE quintile** |  |  |  |  |  |  |  |
| Poorest |  | Ref. |  |  |  |  |  |
| Poorer |  | 0.89(0.79,1.01) |  |  |  |  |  |
| Middle |  | 0.79*(0.7,0.9) |  |  |  |  |  |
| Richer |  | 0.72*(0.63,0.82) |  |  |  |  |  |
| Richest |  | 0.64*(0.55,0.74) |  |  |  |  |  |
| **Religion** |  |  |  |  |  |  |  |
| Hindu |  | Ref. |  |  |  |  |  |
| Muslim |  | 1.03(0.9,1.18) |  |  |  |  |  |
| Christian |  | 0.97(0.81,1.17) |  |  |  |  |  |
| Others |  | 0.83(0.68,1.02) |  |  |  |  |  |
| **Caste** |  |  |  |  |  |  |  |
| Scheduled Caste |  | Ref. |  |  |  |  |  |
| Scheduled Tribe |  | 1.49*(1.28,1.73) |  |  |  |  |  |
| Other Backward Class |  | 0.79*(0.70,0.90) |  |  |  |  |  |
| Others |  | 0.80*(0.70,0.92) |  |  |  |  |  |
| **Place of residence** |  |  |  |  |  |  |  |
| Urban |  | Ref. |  |  |  |  |  |
| Rural |  | 0.53*(0.47,0.59) |  |  |  |  |  |
| **Region** |  |  |  |  |  |  |  |
| North |  | Ref. |  |  |  |  |  |
| Central |  | 0.81*(0.7,0.95) |  |  |  |  |  |
| East |  | 0.85*(0.73,0.98) |  |  |  |  |  |
| Northeast |  | 0.94(0.78,1.12) |  |  |  |  |  |
| West |  | 1.23*(1.05,1.45) |  |  |  |  |  |
| South |  | 0.81*(0.7,0.94) |  |  |  |  |  |
| **Reduce the size of your meals # Place of residence** |  |  |  |  |  |  |  |
| Yes # urban |  |  | Ref. |  |  |  |  |
| No # urban |  |  | 0.80(0.53,1.22) |  |  |  |  |
| No # rural |  |  | 1.54*(1.02,2.32) |  |  |  |  |
| Yes # rural |  |  | 1.57*(1.06,2.46) |  |  |  |  |
| **Don't eat enough food of your choice # Place of residence** |  |  |  |  |  |  |  |
| Yes # urban |  |  |  | Ref. |  |  |  |
| No # urban |  |  |  | 1.03(0.85,1.24) |  |  |  |
| No # rural |  |  |  | 1.72*(1.45,2.01) |  |  |  |
| Yes # rural |  |  |  | 2.26*(1.91,2.66) |  |  |  |
| **Hungry but didn’t eat # Place of residence** |  |  |  |  |  |  |  |
| Yes # urban |  |  |  |  | Ref. |  |  |
| No # urban |  |  |  |  | 0.48*(0.31,0.76) |  |  |
| No # rural |  |  |  |  | 0.94(0.61,1.46) |  |  |
| Yes # rural |  |  |  |  | 1.11(0.74,1.76) |  |  |
| **Not eat for a whole day # Place of residence** |  |  |  |  |  |  |  |
| Yes # urban |  |  |  |  |  | Ref. |  |
| No # urban |  |  |  |  |  | 0.75(0.46,1.26) |  |
| No # rural |  |  |  |  |  | 1.44(0.89,2.39) |  |
| Yes # rural |  |  |  |  |  | 1.37(0.84,2.35) |  |
| **Lost weight due to lack of food # Place of residence** |  |  |  |  |  |  |  |
| Yes # urban |  |  |  |  |  |  | Ref. |
| No # urban |  |  |  |  |  |  | 0.92(0.58,1.52) |
| No # rural |  |  |  |  |  |  | 1.75*(1.12,2.87) |
| Yes # rural |  |  |  |  |  |  | 1.77*(1.08,2.88) |
| Ref: Reference; AOR: Adjusted odds ratio; UOR: Unadjusted odds ratio; #: Interaction effect; Model-2 to model-7 was adjusted for food security, individual, health and household factors. | | | | | | | |
